# Foetal vascular responses to thromboxane receptor blockade

**DOI:** 10.1155/S0962935192000048

**Published:** 1992

**Authors:** B. A. Meyer, J. E. Dickinson, S. W. Walsh, V. M. Parisi

**Affiliations:** 1 Department of Obstetrics, Gynecology and Reproductive Sciences and Departments of Pediatrics, Physiology and Cell Biology University of Texas Medical School Houston USA; 2 St. Luke's Perinatal Center 4400 Wornall Road Kansas City MO 64111 USA

## Abstract

We hypothesized that foetal administration of SQ-29,548, a putative thromboxane receptor blocker, would prevent foeto–placental vasoconstriction produced by the thromboxane mimic U46619. Arterial blood gases, continuous monitoring of maternal and foetal heart rates and blood pressures were performed in chronically catheterized pregnant ewes. Foetal blood flows and vascular resistance were determined with radioactive microspheres. SQ-29,548 effectively blocked the expected vasoconstrictive effects of thromboxane. However, prolonged infusion of SQ-29,548 resulted in significant decreases in umbilical–placental blood flow and foetal mean arterial pressure. This was accompanied by a respiratory acidemia. Potential therapy for the vasoconstrictive disorders of pregnancy with SQ-29,548 awaits further investigation of its intrinsic vasoactive properties in the umbilical–placental vasculature.


Mediators of Inflammation 1, 15–21 (1992)

### Foetal vascular responses to thromboxane receptor blockade

B.A. Meyer,* CA J.E. Dickinson, S.W. Walsh, and V.M. Parisi

Department of Obstetrics, Gynecology and Reproductive Sciences and Departments of Pediatrics, Physiology and Cell Biology, University of Texas Medical School, Houston
*St. Luke's Perinatal Center, 4400 Wornall Road, Kansas City, MO 64111, USA

CA Corresponding Author

**We hypothesized that foetal administration of SQ-29,548, a putative thromboxane receptor blocker, would prevent foeto–placental vasoconstriction produced by the thromboxane mimic U46619. Arterial blood gases, continuous monitoring of maternal and foetal heart rates and blood pressures were performed in chronically catheterized pregnant ewes. Foetal blood flows and vascular resistance were determined with radioactive microspheres. SQ-29,548 effectively blocked the expected vasoconstrictive effects of thromboxane. However, prolonged infusion of SQ-29,548 resulted in significant decreases in umbilical–placental blood flow and foetal mean arterial pressure. This was accompanied by a respiratory acidemia. Potential therapy for the vasoconstrictive disorders of pregnancy with SQ-29,548 awaits further investigation of its intrinsic vasoactive properties in the umbilical–placental vasculature.**

**Key words:** Foetal blood flow, Placental blood flow, SQ-29,548, Thromboxane receptor blockade, U46619

### Introduction

The arachidonic acid metabolite thromboxane (TX) causes peripheral vasoconstriction in the foetus and decreased utero–placental blood flow. These effects are balanced by the vasodilatory actions of prostacyclin (PGI). The normal term human placenta produces approximately equal amounts of TX and PGI. In contrast, in preeclampsia the placental production of TX is increased three-fold while PGI production is less than half of normal.[1] This eicosanoid imbalance is central to current concepts of the pathophysiology of preeclampsia. This imbalance favours the vasoconstrictive effects of TX both peripherally and in the umbilical–placental circulation,[2] and has been postulated to account for the clinical manifestations of the disease.[1] Potential therapy for this disorder should therefore be directed at correcting the TX/PGI imbalance. Potential methods to accomplish this include: increasing PGI production, inhibition of TX synthesis or blockade of the biological actions of TX. PGI administration has been evaluated in the maternal and foetal compartments without foetal benefit. In preeclamptic women, the clinical condition improves, although foetal distress and foetal demise after maternal PGI administration have been reported.[3] Additionally, *in vivo* laboratory experiments have demonstrated significant reductions in umbilical–placental blood flow occur when PGI is administered to the vasoconstricted ovine foetus.[4,5] TX synthesis in the placenta *in vitro* can be reduced by the administration of low-dose aspirin[6,7] and clinical trials have investigated its effectiveness in preeclampsia.[8,9] Evidence exists that there is an increase in TX in the foetal compartment in pregnancies complicated by preeclampsia.[1,10,11] As obstetricians now have direct access to the foetal circulation, and previous investigations have not focused on inhibition of TX effects, this remains a potential foetal therapy. The effects of SQ-29,548 (SQ), a putative TX receptor blocker, have been evaluated in the guinea-pig trachea,[12] rat aorta,[12] rabbit vas deferens,[13] canine coronary artery[14] and swine pulmonary vasculature.[15] These studies have demonstrated SQ to be a pharmacological TX antagonist via blockade of its vasoactive effects. We hypothesized that TX receptor blockade with SQ would prevent the foeto–placental vasoconstriction caused by TX.

### Materials and Methods

Nine pregnant western-bred ewes were studied between 126 and 132 days of gestation. Preoperatively, the animals were given sodium pentothal, 10 mg $kg^{-1}$ i.v., and atropine, 0.6 mg, i.m. This was supplemented by inhalant fluothane during the surgical procedure. Polyvinyl catheters were placed into branches of the maternal femoral artery and vein, and advanced 25 cm to lie in the abdominal aorta and vena cava distal to the renal artery and vein, respectively. The maternal abdomen was opened through a midline incision and a foetal hindlimb was exteriorized through an incision in the uterine wall. An incision was made in the ventral surface of the hindlimb to expose the

© 1992 Rapid Communications of Oxford Ltd femoral artery and vein. Polyvinyl catheters were inserted into these vessels and advanced to lie distal to the renal artery and vein, respectively. The uterine incision was closed, and a foetal forelimb was exteriorized through a separate uterine incision. An incision was made in the dorsal surface and polyvinyl catheters inserted in the axillary artery and vein. These catheters were advanced to lie in the aortic arch and superior vena cava, respectively. An amniotic fluid catheter was anchored to the interior aspect of the uterine wall. The foetus was given 250 mg of chloramphenicol via a foetal vein, and aqueous penicillin G, 600 000 units was injected into the amniotic cavity. All catheters were filled with heparin 1 000 U/ml. Foetal limb and uterine incisions were closed with 3-0 silk and the abdominal incision was closed with 0 polyglycolic acid (Dexon) sutures. The foetal and maternal catheters were tunneled subcutaneously and secured in a pouch on the maternal flank. All skin incisions were closed with surgical staples. The animals were maintained in accordance with the NIH Guide for the Care and Use of Laboratory Animals (NIH Publication No. 80–23, 1978) in the Animal Care Center of the University of Texas Medical School at Houston. The experimental protocol was approved by our institutional Animal Welfare Committee (AWC–MS–86–046). The animals were allowed to recover for 5–7 days after the operation, at which time normal maternal and foetal arterial blood gas values were observed. Studies were conducted with ewes standing quietly in metabolic cages with unlimited access to food and water. Maternal and foetal mean arterial pressures and foetal venous pressure and amniotic cavity pressure were recorded continuously with an R611 Beckman physiological recorder (Schiller Park, IL) and Statham P23Db transducers (Spectramed, Oxnard, CA). The foetal heart rate was determined using a cardiotachometer triggered from the pulse pressure wave in the foetal ascending aorta. Maternal and foetal distal aortic arterial pH, $PO_2$, $PCO_2$, bicarbonate ($HCO_3^-$) and base excess (BE) were measured with a Corning 158 blood gas analyser (Medfield, MA). Temperatures of 37°C and 39°C, respectively, were utilized to measure maternal and foetal blood gas parameters. Foetal haemoglobin concentrations were measured with an OSM-2 hemoximeter (London Company, Cleveland, OH). Maternal and foetal serum lactates were measured enzymatically (Sigma Diagnostics, St. Louis, MO). Foetal regional blood flow was measured by the radioactive microsphere technique as previously described.[16] Approximately $10^6$ microspheres, 15 $\mu$m in diameter, labelled with Strontium$^{85}$, Scandium$^{46}$, Tin$^{113}$ or Cobalt$^{57}$ (3M Company, St. Paul, MN and New England Nuclear, DuPont Diagnostics-Imaging, North Billerica, MA), were injected into the foetal vena cava over 15–30 s. Simultaneously, an integrated arterial reference sample was withdrawn from both of the foetal arterial catheters at 2.06 ml min$^{-1}$ for 2 min beginning coincidentally with the microsphere injection. The order of isotope administration was altered with each experiment to avoid sequencing.

*Experimental design:* SQ-29,548 (E.R. Squibb and Sons, Princeton, New Jersey) was dissolved in 95% ethanol (10 mg ml$^{-1}$), and further diluted with 9 vol of 2 mM $Na_2CO_3$. This solution was freshly prepared the morning of each experiment. Prior to the microsphere studies, in a separate series of experiments, the ethanol/$Na_2CO_3$ vehicle was infused for 120 min via the foetal vena cava in six animals. Equivalent volumes to the subsequent SQ bolus and infusion were used to evaluate potential intrinsic vasoactivity of the vehicle. No foetus received more than 30 ml of infusate during any individual experiment. Continuous recording of maternal and foetal arterial pressures and heart rates was performed. Maternal and foetal distal aortic arterial blood gases were measured at 15 min intervals. The animals were then allowed to recover for a minimum of 24 h before blood flow experimentation. Prior to the blood flow portion of each experiment, maternal and foetal arterial pressures and heart rates were continuously recorded. As the active metabolite of TX ($TXA_2$) is extremely labile, it is necessary to use the stable analogue U46619 for experimental studies. After normal maternal and foetal arterial blood gases were demonstrated, the TX mimic U46619 (Upjohn, Kalamazoo, MI) at a concentration of 5 $\mu$g ml$^{-1}$ in sterile 0.9% saline, was infused into the foetal vena cava at a rate of 5 $\mu$g min$^{-1}$. Maternal and foetal arterial blood gases and serum lactates were obtained after 20 min of infusion to document vasoactivity. This dosage would be expected to produce foetal hypertension, bradycardia and significant reductions in arterial pH.[5] The animals were allowed to recover for at least 2 h before further experimentation. A return to baseline values in maternal and foetal arterial blood gases, heart rates and mean arterial pressures was documented prior to beginning the blood flow portion of the experiment. Maternal and foetal arterial pressures and heart rates were again continuously recorded. Maternal and foetal haemoglobin concentrations were obtained after each microsphere injection. Maternal and foetal arterial blood gases, serum lactates and foetal blood flows were measured in the control period. A bolus injection of 1.0 mg of SQ was given via the foetal vena cava over a 1 min period. An infusion of SQ at 0.2 mg kg foetus$^{-1}$ h$^{-1}$ was then begun. Maternal and foetal arterial blood gases, serum lactates and foetal blood flows were assessed 20 min after commencement of the SQ infusion (SQ). TX mimic (TX) was then infused into the foetal vena cava at a dose of 5 $\mu$g min$^{-1}$ while maintaining the SQ infusion. All measurements were repeated 20 min after adding the TX to the SQ infusion (SQ + TX). The TX infusion was then discontinued. The SQ infusion was continued and all measurements were then repeated 30 min later (SQ + 70). Maternal and foetal arterial blood gases and serum lactates were repeated after a further 30 min of SQ infusion (SQ + 100).

*Radioactive microsphere analysis:* After each experiment was completed, the animals were killed with an 10 ml i.v. injection of T-61 Euthanasia (Hoechst-Roussel, Agri-Vet, Sommerville, NJ). The foetal brain, heart, kidneys, small and large intestine, hindlimb and upperlimb skeletal muscle and placental cotyledons were dissected from other foetal tissues. Appropriate intravascular placement of all catheters was verified at autopsy. All tissues were ashed and radioactivity was assayed with a Nuclear Data convertible multichannel analyser (Schaumburg, IL) connected to a Tracor sodium iodide crystal detector and sample changer (Chicago, IL). Known standards of each isotope were assayed simultaneously. The number of microspheres in, and blood flow to, each organ was calculated by modification of a peak search program developed by Nuclear Data for our laboratory. Blood flow to each organ was calculated using the arterial reference sample appropriate to its anatomical location. Vascular resistance was defined as mean arterial pressure minus venous pressure minus amniotic cavity pressure divided by blood flow to an organ, and expressed as mmHg ml$^{-1}$ min. Amniotic cavity pressure was subtracted from both mean arterial and venous pressure prior to calculation of the vascular resistance. Umbilical placental blood flow was expressed as ml min$^{-1}$ kg$^{-1}$ foetal weight of tissue. Brain, cardiac, renal, intestinal and skeletal muscle blood flow was expressed as ml min$^{-1}$ 100 gm$^{-1}$ of tissue. All organs studied and all arterial reference samples contained greater than 400 microspheres and thereby satisfied criteria for accuracy of blood flow measurements within $\pm$10% by the radioactive microsphere technique.[17]

*Statistical analysis.* Statistical analysis was done by two-way analysis of variance with random block and paired *t*-test as appropriate, using computer statistical software (Abstat, Anderson-Bell, Parker, Co.). Logarithmic transformation [log($x$ + 1)] was used when variances were not equal. A probability of $p < 0.05$ was chosen to represent statistical significance. All data are expressed as mean $\pm$ SE.

### Results

Maternal heart rate, mean arterial pressure, arterial blood gas parameters and blood lactate levels were not significantly altered at any time during experimentation. Similarly, no significant changes in amniotic cavity or foetal venous pressure were observed during any study period. There were no alterations in maternal arterial blood gas parameters, or foetal haemoglobin concentrations at any time during experimentation.

*Vehicle infusion:* No significant changes in foetal or maternal cardiorespiratory parameters were observed during infusion of the ethanol/$Na_2CO_3$ vehicle over the 120 min infusion period. The prolonged vehicle infusion time was chosen due to its technical ease, and to ensure that the ethanol/$Na_2CO_3$ vehicle exhibited no cumulative intrinsic vasoactivity with extended administration. Foetal heart rate varied from 164 $\pm$ 4 beats per min (bpm) at the control time period, to 167 $\pm$ 5 bpm at 120 min (range: 157 $\pm$ 8 to 174 $\pm$ 9 bpm). Foetal mean arterial pressure went from 39.4 $\pm$ 2.2 at control, to 40.6 $\pm$ 2.5 mmHg at 120 min (range: 37.9 $\pm$ 3.7 to 42.5 $\pm$ 1.7). Control arterial pH was 7.40 $\pm$ 0.02, with 7.39 $\pm$ 0.01 observed after 120 min (range: 7.38 $\pm$ 0.02 to 7.41 $\pm$ 0.02). There were no statistically significant differences in the above measurements during the 120 min evaluation period ($p > 0.20$). Similarly, there were no statistically significant changes in foetal arterial $pO_2$, $pCO_2$, $HCO_3^-$ and BE throughout the vehicle infusion ($p > 0.20$).

*U46619 effects.* Foetal bradycardia and hypertension were demonstrated in response to the test dose of i.v. U46619 at 5 $\mu$g min$^{-1}$. Foetal heart rate fell from 167 $\pm$ 4 to 131 $\pm$ 13 bpm ($p < 0.05$) (Fig. 1), and mean arterial pressure rose from 38.7 $\pm$ 1.4 to 48.6 $\pm$ 2.0 mmHg ($p < 0.05$) (Fig. 1). Foetal heart rate was not significantly altered from control values at any other time during the study. The test dose of TX mimic U46619 caused a significant deterioration in foetal acid-base status. This was demonstrated by significant reductions in pH, $pO_2$ and BE, from 7.39 $\pm$ 0.02 to 7.33 $\pm$ 0.03, 26.7 $\pm$ 2.1 to 22.8 $\pm$ 2.6 torr, and 2.5 $\pm$ 1.8 to $-1.9 \pm 2.6$, respectively ($p < 0.05$). This was accompanied by significant elevations in $pCO_2$ from 42.8 $\pm$ 2.1 to 48.3 $\pm$ 1.5 torr ($p < 0.05$). Bicarbonate was not significantly altered by the test infusion of TX mimic. Analysis of foetal blood lactates revealed a significant increase after administration of U46619 from 31.2 $\pm$ 3.6 to 38.3 $\pm$ 2.9 mg dl$^{-1}$ ($p < 0.05$) (Fig. 2).

*SQ-29,548 infusion:* No significant alterations in foetal heart rate or arterial pressures were noted after 20 min of SQ infusion. However, a significant decrease

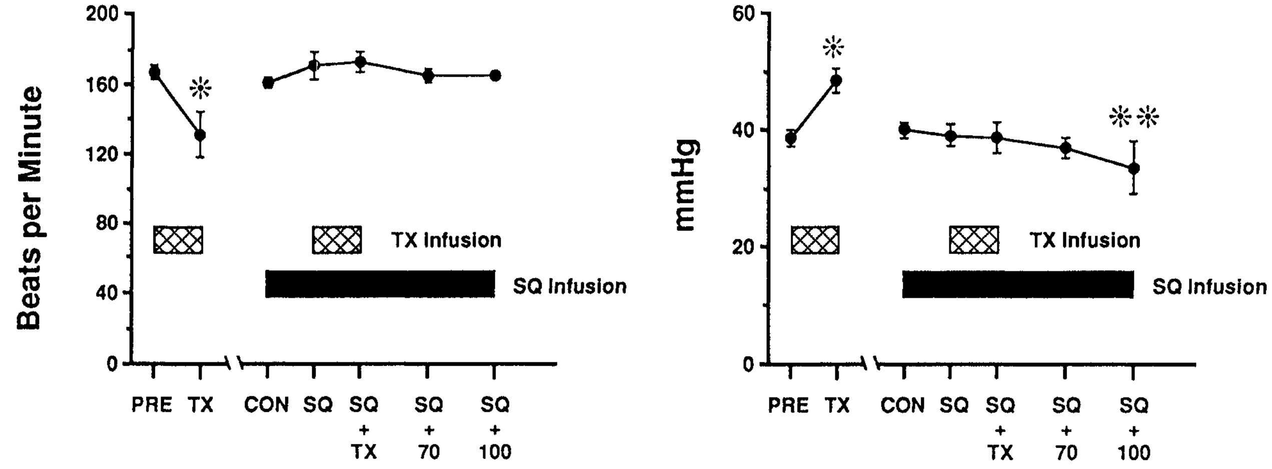

FIG. 1. Foetal heart rate in the left panel, and foetal mean arterial pressure in the right panel. PRE: baseline time period; TX: text TX mimic time period; CON: control time period; SQ: 20 min of SQ infusion; SQ + TX: simultaneous SQ and TX mimic infusion; SQ + 70: 70 min of SQ infusion; SQ + 100: 100 min in total of SQ infusion. All data are mean ± SE, $n = 9$. *$p < 0.05$ vs. PRE; **$p < 0.05$ vs. CON.

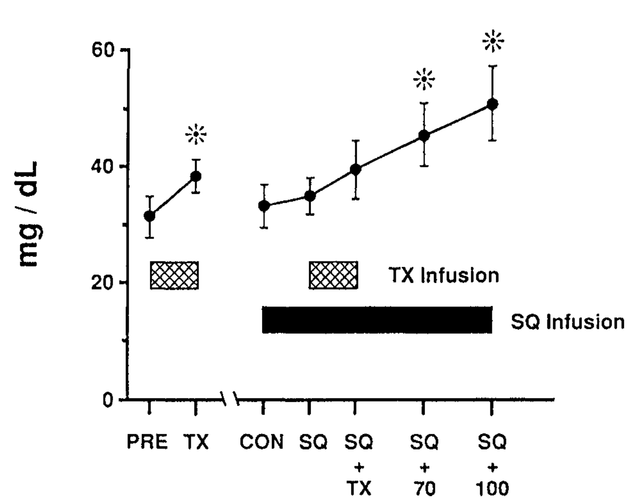

FIG. 2. Foetal blood lactates. PRE: baseline time period; TX: test TX mimic time period; CON: control time period; SQ: 20 min of SQ infusion; SQ + TX: simultaneous SQ and TX mimic infusion; SQ + 70: 70 min of SQ infusion; SQ + 100: 100 min in total of SQ infusion. All data are mean ± SE, $n = 8$. *$p < 0.05$ vs. control (CON).

in foetal mean arterial pressure from 40.0 ± 1.4 at control time period (CON) to 33.5 ± 4.5 mmHg was present after 100 min of SQ infusion (SQ + 100) (Fig. 1). During the blood flow portion of the experiment, foetal arterial blood gases were not significantly altered from control values after 20 min of SQ infusion (SQ) (Table 1). However, after 70 and 100 min of SQ administration, significant decreases in foetal pH and $pO_2$ were observed, from 7.41 ± 0.02 to 7.36 ± 0.01 and 26.5 ± 3.0 to 21.7 ± 2.3 torr, respectively. Foetal $pCO_2$ was significantly elevated after 100 min of SQ infusion from 45.9 ± 2.0 to 48.9 ± 2.0 torr. There were no changes in foetal $HCO_3^-$ or BE during the microsphere studies (Table 1). Umbilical–placental blood flow was not altered from control levels (CON) after 20 min of SQ infusion (SQ) (Fig. 3). Unexpectedly, umbilical–placental blood flow was significantly reduced from the control value of 292.3 ± 32.4 to 205.1 ± 22.3 ml $min^{-1}$ $kg^{-1}$ foetal weight after 70 min of SQ infusion (SQ + 70) ($p < 0.05$). This reduction in umbilical–placental blood flow was significantly different from the control and SQ values, and was temporally related to the changes in foetal acid-base status. Similarly, umbilical–placental vascular resistance was not altered after 20 min of SQ infusion (Fig. 4). However, umbilical–placental vascular resistance was significantly increased after 70 min of SQ infusion from 0.15 ± 0.02 to 0.20 ± 0.02 mmHg $ml^{-1}$ min kg foetal weight. Blood flow and resistance to specific foetal organ vascular beds was evaluated. Blood flow to the brain, heart, kidney, intestine and skeletal muscle was not

**Table 1.** Foetal arterial blood gases

| | pH | $PO_2$(torr) | $PCO_2$(torr) | $HCO_3^-$(meq/l) | BE |
|---|---|---|---|---|---|
| CON | 7.41 ± 0.02 | 26.5 ± 3.0 | 45.9 ± 2.0 | 27.4 ± 1.3 | 3.1 ± 1.2 |
| SQ | 7.40 ± 0.02 | 26.7 ± 3.1 | 42.9 ± 2.9 | 26.2 ± 1.8 | 2.4 ± 1.6 |
| SQ + TX | 7.38 ± 0.01 | 25.3 ± 3.6 | 44.1 ± 2.4 | 25.5 ± 1.8 | 1.3 ± 1.7 |
| SQ + 70 | 7.37 ± 0.01* | 22.4 ± 1.9* | 46.5 ± 2.3 | 26.4 ± 1.5 | 2.2 ± 1.5 |
| SQ + 100 | 7.36 ± 0.01* | 21.7 ± 2.3* | 48.9 ± 2.0* | 25.9 ± 1.5 | 2.7 ± 1.7 |

CON: control time period; SQ: 20 min of SQ infusion; SQ + TX: simultaneous SQ and TX mimic infusion; SQ + 70: 70 min of SQ infusion; SQ + 100: 100 min in total of SQ infusion. All data are mean ± SE; $n = 8$; $p < 0.05$ vs. control.

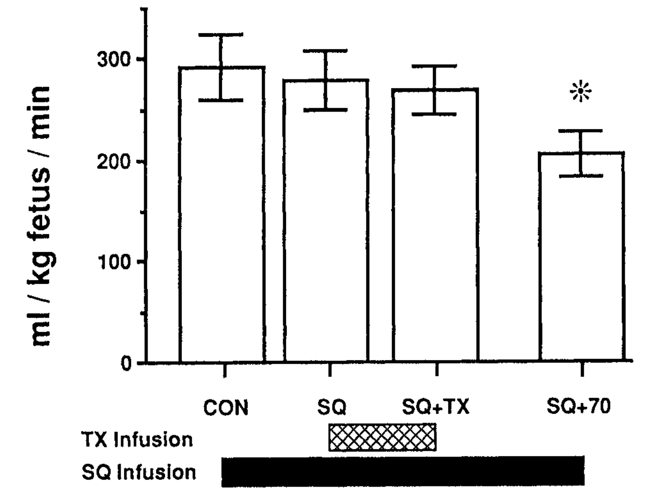

FIG. 3. Foetal placental blood flow responses in ml $min^{-1}$ $kg^{-1}$ foetal weight. CON: control time period; sQ: 20 min of SQ infusion; SQ + TX: simultaneous SQ and TX mimic infusion; SQ + 70: 70 min of SQ infusion. All data are mean ± SE, $n = 8$. *$p < 0.05$ vs. CON.

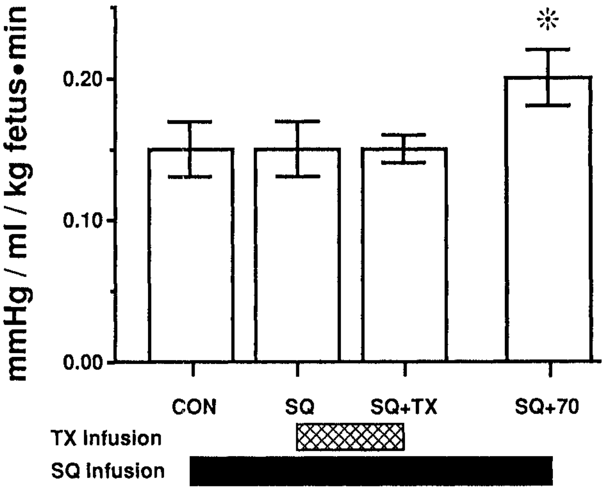

FIG. 4. Foetal placental vascular resistance responses in mmHg $ml^{-1}$ min $kg^{-1}$ foetal weight. CON: control time period; SQ: 20 min of SQ infusion; SQ + TX: simultaneous SQ and TX mimic infusion; SQ + 70: 70 min of SQ infusion. All data are mean ± SE, $n = 8$. *$p < 0.05$ vs. CON.

significantly altered at any of the time periods analysed (Table 2). Foetal blood lactate progressively increased throughout the experimental time period, with significant elevations from control values after 70 and 100 min of SQ administration (33.1 ± 3.7, 45.5 ± 5.5 and 50.7 ± 6.3 mg $dl^{-1}$, respectively) ($p < 0.05$) (Fig. 2).

*SQ-29,548/U46619 simultaneous infusion:* The previously demonstrated foetal hypertension and bradycardia caused by TX were blocked by SQ (Fig. 1). Additionally, the alterations in foetal acid-base status caused by the TX mimic were absent (Table 1). The expected decrease in umbilical–placental blood flow during TX administration (SQ + TX) was effectively blocked (Fig. 3). The expected increase in placental vascular resistance with TX administration did not occur (Fig. 4). Indeed, the reported decrease in blood flow to the renal, gastrointestinal and skeletal muscle vascular beds which has been previously reported with this dosage of TX was not observed (Table 2) (Parisi VM, Walsh SW, Stockmar EJ. Foetal placental vascular responses to prostacyclin after thrombox-

**Table 2.** Foetal organ blood flows

| | CON | SQ | SQ + TX | SQ + 70 |
|---|---|---|---|---|
| Brain | 129.0 ± 16.9 | 140.3 ± 17.0 | 134.1 ± 16.1 | 137.8 ± 21.3 |
| Kidney | 199.1 ± 10.3 | 190.9 ± 7.6 | 188.4 ± 14.8 | 176.4 ± 14.3 |
| Skeletal muscle | 20.0 ± 4.2 | 16.8 ± 2.0 | 14.3 ± 2.3 | 16.4 ± 2.6 |
| Intestine | 67.2 ± 9.0 | 76.1 ± 13.1 | 66.9 ± 7.9 | 67.6 ± 9.9 |
| Heart | 218.8 ± 16.2 | 235.5 ± 13.9 | 214.4 ± 30.8 | 217.5 ± 27.4 |

CON: control time period; SQ: 20 min of SQ infusion; SQ + TX: simultaneous SQ and TX mimic infusion; SQ + 70: 70 min of SQ infusion. All data are mean ± SE; $n = 8$; all vaues not significantly different from control.

**Table 3.** Calculated foetal organ vascular resistance

| | CON | SQ | SQ + TX | SQ + 70 |
|---|---|---|---|---|
| Brain | 0.34 ± 0.05 | 0.30 ± 0.03 | 0.33 ± 0.06 | 0.32 ± 0.05 |
| Kidney | 0.21 ± 0.01 | 0.21 ± 0.02 | 0.22 ± 0.03 | 0.23 ± 0.02 |
| Skeletal muscle | 2.82 ± 0.53 | 2.68 ± 0.42 | 3.28 ± 0.53 | 3.24 ± 0.58 |
| Intestine | 0.70 ± 0.11 | 0.61 ± 0.08 | 0.64 ± 0.07 | 0.64 ± 0.09 |
| Heart | 0.19 ± 0.02 | 0.16 ± 0.01 | 0.19 ± 0.03 | 0.19 ± 0.03 |

CON: control time period; SQ: 20 min of SQ infusion; SQ + TX: simultaneous SQ and TX mimic infusion; SQ + 70: 70 min of SQ infusion. All data are mean ± SE; $n = 8$; all vaues not significantly different from control.

ane and angiotensin II induced vasoconstriction. Presented at the 34th annual meeting of the Society of Gynecologic Investigation, Atlanta, Georgia, March 18–21, 1987, Abstract #360). Similarly, the expected increase in vascular resistance to the kidneys, intestine and skeletal muscle was not present with simultaneous SQ and TX administration (Table 3).

### Discussion

The finding of an imbalance in TX/PGI production by the human placenta in preeclampsia[1] has stimulated discussion regarding potential therapy for this disease. Previous human research had focused on correcting the deficiency in PGI by increasing PGI.[3] Subsequent investigators have evaluated inhibition of thromboxane production both *in vivo*[8,9] and *in vitro*.[6,7] The foetus may be adversely affected by the increase in thromboxane production found in pregnancies complicated by preeclampsia. Since thromboxane is a potent vasoconstrictor in the ovine foetus,[5,18] we investigated modulation of thromboxane vasoactivity as a potential *in utero* foetal therapy. Short term foetal administration of SQ over 20 min did not result in any significant alterations in foetal heart rate, mean arterial pressure or arterial blood gas parameters. No changes in blood flow or vascular resistance were demonstrated in the umbilical–placental vasculature. The vascular circulations of the brain, heart, kidneys, intestine and skeletal muscle were also not affected by 20 min of SQ infusion. Thus, foetal administration of SQ over 20 min does not exhibit intrinsic vasoactivity. Subsequently, however, a significant decrease in foetal mean arterial pressure was present at SQ + 100. This change may represent blockade of endogenous foetal TX, resulting in a decrease in intrinsic vascular tone. These alterations may be due to a specific effect of SQ, or may occur with any thromboxane antagonist.

The vasoactivity of the thromboxane mimic U46619 was demonstrated in our study by significant alterations in foetal cardiorespiratory parameters. These effects on foetal heart rate, mean arterial pressure and arterial blood gas parameters were prevented by foetal administration of SQ. This was evidenced by the lack of any significant changes in cardiorespiratory measurements when these compounds were simultaneously infused. Similarly, the vasoconstriction in the umbilical–placental circulation previously demonstrated with foetal thromboxane administration,[5] was blocked by SQ. There were no significant alterations in blood flow or vascular resistance in any of the foetal organs examined when U46619 and SQ were administered simultaneously. Indeed, the expected thromboxane-induced vasoconstriction in the renal,[5] gastrointestinal and skeletal muscle vasculature[18] was prevented by SQ. These data would appear to support the actions of SQ as a thromboxane receptor blocker.

Previous investigations have reported that the vasoactive effects of U46619 resolve within 30 min after cessation of foetal infusion (Parisi VM, Stockmar EJ. Foetal cardiorespiratory responses to prostacyclin and thromboxane. Presented at the 7th annual meeting of the Society for Perinatal Obstetricians, Orlando, Florida, February 2–5, 1987, Abstract #109). Thus, the umbilical–placental vasoconstriction observed 30 min after the discontinuation of the TX mimic infusion (i.e. after a total of 70 min of SQ infusion) was an unexpected finding. This umbilical–placental vasoconstriction was not accompanied by alterations in blood flow or vascular resistance in the other foetal organs examined. The respiratory alterations in arterial blood gas parameters which we observed after 70 and 100 min of SQ infusion, are consistent with this decrease in umbilical–placental perfusion. The progressive rise in foetal blood lactate is also consistent with the observed decrease in umbilical–placental perfusion. The 25% decrease in skeletal muscle blood flow may be the origin of this metabolic product. The lack of statistical significance ($p = 0.11$) may be due to the relatively small number of animals. Additionally, decreased placental clearance of lactate due to the vasoconstriction in the umbilical–placental circulation may account for the significant increases in foetal blood lactate levels during the experimental period.

Our finding of umbilical–placental vasoconstriction after 70 min of SQ infusion is in conflict with its putative action as a thromboxane receptor blocker.[12] We speculate that SQ may possess activity as a weak thromboxane agonist in the umbilical–placental circulation. This is consistent with our findings of the absence of short term vasoactivity, effective thromboxane blockade, and subsequent vasoconstriction. In this situation, the lack of changes in perfusion to other organs in the face of decreased umbilical–placental blood flow may be due to small reductions in flow throughout all foetal tissues or to decreases in perfusion to specific organs which were not examined. Decreased metabolic clearance of SQ would result in umbilical–placental vasoconstriction if it is indeed a weak thromboxane agonist. The speculation that SQ acts as a weak thromboxane agonist does not explain the significant decrease in foetal mean arterial pressure at SQ + 100. The formation and action of a vasoactive metabolite of SQ could represent an alternative explanation for the temporal delay in umbilical–placental vasoconstriction.

Another speculation which may account for the observed alteration in foeto–placental blood flow is passive vasoconstriction of the umbilical–placental circulation based on Poiseuille's law. Poiseuille's law states that vascular resistance is equal to the pressure drop across a vascular bed (arterial — venous pressure) divided by the rate of blood flow to an organ. This pressure differential is usually estimated by foetal mean aortic blood pressure. Blood flow to an organ is related to the length and radius of the afferent vessel and the viscosity of blood. Since the placenta is the organ furthest from the heart, blood flow to the placenta must overcome the greater resistance created by this increased distance.[19] We speculate that SQ may cause passive vasoconstriction as well as thromboxane receptor blockade. In this setting, blood flow would take the path of least resistance and be preferentially diverted to organs more proximal to the heart than the placenta. Since the lung is the most proximal organ to the heart, and other investigators have demonstrated pulmonary responsiveness to SQ,[15] this organ may be the recipient of increased blood flow. According to Poiseuille's law, an increase in arterial pressure accompanied by a decrease in blood flow represents active vasoconstriction. In contrast, the significant reduction in umbilical–placental perfusion which we observed represents passive rather than active vasoconstriction, since foetal mean arterial pressure and umbilical–placental blood flow both decreased. The foetal mean arterial pressure at SQ + 70 was decreased, although these changes did not achieve statistical significance until 100 min of SQ infusion. It is likely that the lack of significance at SQ + 70 is due to the relatively small number of animals in our study. Based on the arterial blood gas findings, it is reasonable to assume that umbilical–placental blood flow did not improve at SQ + 100. Thus, based on Poiseuille's law, SQ may possess intrinsic vasoactivity which results in passive vasoconstriction of the umbilical–placental vasculature.

We conclude that SQ blocks the foetal vascular response to thromboxane mimic. However, prolonged infusion of SQ results in a progressive deterioration in the foetal acid-base status and significant reductions in umbilical–placental blood flow. The physiological mechanism responsible for these findings remains unclear, and may apply to other thromboxane antagonists. The clinical relevance of these data lies in the potential for therapy of the vasoconstrictive disorders of pregnancy associated with an imbalance in the production of TX/PGI.

### References

1. Walsh SW. Preeclampsia: an imbalance in placental prostacyclin and thromboxane production. *Am J Obstet Gynecol* 1985; **152:** 335–340.
2. Ylikorkala O, Viinikka L. Thromboxane A2 in pregnancy and puerperium. *Br Med J* 1980; **281:** 1601–1609.
3. Lewis PJ, Shepard GL, Ritter J, *et al.* Prostacyclin and preeclampsia. *Lancet* 1981; **1:** 559–563.
4. Parisi VM, Walsh SW. Fetal placental vascular responses to prostacyclin after angiotensin II induced vasoconstriction. *Am J Physiol* 1989; **257(20):** 102–107.
5. Parisi VM, Walsh SW. Feto-placental vascular responses to prostacyclin after thromboxane induced vasoconstriction. *Am J Obstet Gynecol* 1989; **160:** 502–507.
6. Thorp JA, Walsh SW, Brath PS. Low dose aspirin inhibits thromboxane but not prostacyclin production by human placental arteries. *Am J Obstet Gynecol* 1988; **159:** 1381–1385.
7. Nelson DM, Walsh SW. Aspirin differentially affects thromboxane and prostacyclin production by trophoblast and villus core compartments of human placental villi. *Am J Obstet Gynecol* 1989; **161:** 1593–1598.
8. Schiff E, Peleg E, Goldenberg M, *et al.* The use of aspirin to prevent pregnancy-induced hypertension and lower the ratio of thromboxane A2 to prostacyclin in relatively high risk pregnancies. *New Engl J Med* 1989; **321:** 351–356.
9. Wallenberg HC, Dekker GA, Makovitz JW, Rotmans P. Low-dose aspirin prevents pregnancy-induced hypertension and preeclampsia in angiotensin-sensitive primigravidae. *Lancet* 1986; **1(8471):** 1–3.
10. Ylikorkala O, Makila U-M, Viinikka L. Amniotic fluid prostacyclin and thromboxane in normal, preeclamptic, and some other complicated pregnancies. *Am J Obstet Gynecol* 1981; **141:** 487–491.
11. Takagi S, Den K. Prostacyclin and thromboxane A2 in preeclamptic umbilical circulation. In: Hayaishi O, Yamamoto S, eds. *Advances in Prostaglandin, Thromboxane and Leukotriene Research.* New York; Raven Press, 1985: (15) 619–626.
12. Ogletree ML, Harris DN, Greenberg R, *et al.* Pharmacologic actions of SQ-29,548, a novel selective thromboxane antagonist. *J Pharmacol Exp Ther* 1985; **234(2):** 435–441.
13. Ogletree ML. Overview of physiological and pathophysiological effects of thromboxane A2. *Federation Proc* 1987; **46:** 133–138.
14. Ashton JH, Schmitz JM, Campbell WB, *et al.* Inhibition of cyclic flow variations in stenosed canine coronary arteries by thromboxane A2/prostaglandin H2 receptor antagonists. *Circ Res* 1986; **59:** 568–578.
15. Schumacher WA, Adams HD, Ogletree ML. Effect of the thromboxane A2-receptor antagonists, SQ-29,548 and SQ-28,668, on the pulmonary hypertensive response to endotoxemia in swine. *Pharmacology* 1987; **34:** 301–308.
16. Rudolph AM, Heymann MA. The circulation of the fetus *in utero:* method for studying distribution of blood flow, cardiac output and organ blood flow. *Circ Res* 1967; **21:** 163–184.
17. Buckberg GD, Luck JC, Payne DB, *et al.* Some sources of error in measuring regional blood flow with radioactive microspheres. *J Appl Physiol* 1971; **31:** 598.
18. Trudinger BJ, Connelly AJ, Giles WB, *et al.* The effects of prostacyclin and thromboxane analogue (U46619) on the fetal circulation and umbilical flow velocity wave forms. *J Dev Physiol* 1989; **11(3):** 179–184.
19. Walsh SW, Parisi VM. The role of prostanoids and thromboxane in the regulation of placental blood flow. In: Rosenfeld CR, ed. *The Uterine Circulation.* Ithaca, New York; Perinatology Press, 1989: (14) 274–298.

ACKNOWLEDGEMENTS. We would like to thank Dr. Margaret Poage, Mr. Peter Brath and Ms. Susie Chmielowiec-Bray for their assistance in this project. This project was supported by NIH HD00782 and HD20973 and presented in part at the 10th Annual Meeting of the Society of Perinatal Obstetricians, January 25–27, 1990, Houston, Texas, USA.

**Received 10 October 1991; accepted with revision 27 November 1991**
